# Comprehensive analysis of the oncogenic and immunological role of *SPON2* in human tumors

**DOI:** 10.1097/MD.0000000000035122

**Published:** 2023-09-15

**Authors:** Jiali Tang, Qing Huang, Xuanwen Li, Shinong Gu

**Affiliations:** a College of Environment and Public Health, Xiamen Huaxia University, Xiamen, P.R. China; b Graduate School of Health Science, Suzuka University of Medical Science, Suzuka, Japan.

**Keywords:** cancer, immunological, prognosis, *SPON2*, survival analyses

## Abstract

**Background:**

Sapiens spondin-2 (*SPON2*) is a protein found in the extracellular matrix that plays a role in a number of processes, including immune reactions and cell adhesion, and is closely linked to the emergence of a number of tumor types. However, we know very little about Sapiens spondin-2. Therefore, we performed a systematic pan-carcinogenic analysis to explore the relationship between Sapiens spondin-2 and cancers.

**Materials and methods:**

By comprehensive use of datasets from TCGA, GEO, GTEx, HPA, CPTAC, GEPIA2, TIMER2, cBioPortal, STRING, we adopted bioinformatics methods to dig up the potential carcinogenesis of *SPON2*, including dissecting the correlation between *SPON2* and gene expression, prognosis, gene mutation, Immunohistochemistry staining, immune cell infiltration, and constructed the interaction network of a total of 54 *SPON2*-binding proteins as well as explored the enrichment analysis of *SPON2*-related partners.

**Results:**

The expression of Sapiens spondin-2 in most tumor tissues was higher than that of normal tissues. In addition, *SPON2* showed the early diagnostic value in 33 kinds of tumors and was positively or negatively associated with the prognosis of different tumors. It also validates that *SPON2* is the gene associated with the majority of immune-infiltrating cells in pan-cancer. High *SPON2* expression is associated with tumor progression related pathways.

**Conclusion:**

We found and validated the potential use of *SPON2* in cancer detection for the first time through pan-cancer analysis. The expression levels of *SPON2* in various tumors were quite different from those in normal tissues. Furthermore, the performance of *SPON2* in tumorigenesis and tumor immunity verified our hypothesis. At the same time, it has high specificity and sensitivity in cancer detection. Therefore, *SPON2* can be employed as an auxiliary index for the initial diagnosis of tumors and a prognostic marker for various types of tumors.

## 1. Introduction

*SPON2* is an extracellular matrix protein belonging to the mindin/F-spondin family.^[1]^ *SPON2* includes an N-terminal F-spondin (FS) domain and C-terminal thrombospondin type 1 repeat (TSR). The FS domain mediates the interaction between *SPON2* and integrins, whereas the TSR domain contributes to *SPON2* as a pathogen pattern recognition molecule. Through the above dual role, the FS and TSR domains of *SPON2* promote activation of both adaptive and innate immune responses.^[2]^ Besides this, *SPON2* has been proved to play a part in hippocampal neuronal growth and diabetic nephropathy.^[3,4]^ More importantly, previous studies showed that *SPON2* was overexpressed in breast cancer,^[5]^ gastric cancer,^[6,7]^ prostate cancer,^[8–13]^ Barrett’s adenocarcinoma,^[14]^ pancreatic cancer,^[15]^ ovarian cancer,^[16,17]^ pulmonary adenocarcinoma,^[18]^ and Hepatocellular Carcinoma.^[19–21]^ In addition, *SPON2* has been put forward as a new serum and histological diagnostic biomarker, as well as an independent prognostic indicator for gastric cancer,^[7]^ ovarian cancer^[16,17]^ and prostate cancer.^[10–13]^ Also, it has been claimed that *SPON2* overexpression was found in clear cell renal cell carcinoma (ccRCC) and relevant to tumor stage, Fuhrman grade and recurrence after surgery in patients with localized ccRCC , so it can be served as a predictor of recurrence.^[22]^ Despite extensive clinical data, there is currently no proof of a pan-carcinogen link between *SPON2* and different tumor forms.

The Cancer Genome Atlas (TCGA) project and the Gene Expression Omnibus (GEO) database were used in our investigation, which represents the first pan-cancer characterization of *SPON2*. To investigate the underlying molecular processes of *SPON2* in the etiology or clinical prognosis of various malignancies and clinical significance, we also included a set of variables such as gene expression, survival status, gene modification, immunohistochemistry, immune infiltration, and associated cellular pathways.

## 2. Materials and methods

### 2.1. Gene expression analysis

We entered *SPON2* into the “Gene DE” module of the TIMER2 (Tumor Immune Estimation Resource (version 2)) site (http://timer.cistrome.org/) and looked at the differential in *SPON2* expression between tumor and nearby normal tissues for the various cancers or particular tumor subtypes in the TCGA dataset. For some normal tumor tissues without normal or highly limited [e.g., TCGA-GBM (Glioblastoma multiforme), TCGA-LAML (Acute myeloid leukemia), etc.], we used the “Expression analysis-Boxplot” module of gepia2 (Gene Expression Profiling Interactive Analysis, version 2) network service (http://gepia2.cancer-pku.cn/#analysis) to obtain box plots of the expression differences between these tumor tissues and the corresponding normal tissues of the GTEx (Genotype-Tissue Expression) database, the relative gene expression values were calculated at *P* value cutoff = .01, log2FC (fold change) cutoff = 1 and settings of “match TCGA normal and GTEx data. Furthermore, using the “Pathological staging plots” module of HEPIA2, we acquired violin plots of *SPON2* expression in all TCGA tumors at various pathological stages (stage I, II, III, and IV). The converted expression data is applied to box or violin plots using the formula Log2 [TPM (Transcripts per million) + 1].

The CPTAC (Clinical proteome tumor analysis consortium) dataset can be analyzed for protein expression using the UALCAN portal (http://ualcan.path.uab.edu/index.html), which examines tumor transcriptome data by integrating RNA sequencing (RNA SEQ) and clinical data from 31 different types of cancers.^[23,24]^ We utilized the input “*SPON2*” to choose the datasets of 6 tumors: pancreatic adenocarcinoma (PAAD), Lung squamous cell carcinoma (LUSC), Head and neck squamous carcinoma (HNSC), ccRCC, Glioblastoma multiforme (GBM) and lung adenocarcinoma (LUAD).

### 2.2. Survival prognosis analysis

We were able to get *SPON2* significance map data for overall survival (OS) and disease-free survival (DFS) in all TCGA tumors using the “Survival Particle” module of GEPIA2.^[25]^ Additionally, we divided the cohorts with high and low expression using the cutoff-high (50%) and cutoff-low (50%) expression thresholds. The “Survival Analysis” module of GEPIA2 is used to generate a survival plot for the hypothesis test, which employs a log-rank test. We also calculated the hazard ratio and log-rank *P* values through purity-adjusted Spearman grade correlation test and other statistical test methods

### 2.3. Genetic alteration analysis

We entered “*SPON2*” into the “Quick Select” part of the cBioPortal Web (https://www.cbioportal.org/) after logging in to learn more about the genetically changed properties of *SPON2*. We then chose the “TCGA Pan-Cancer Atlas Studies” option. The “Cancer Type Summary” module showed the frequency of alterations, mutation types, and copy number changes (CNA) data for all TCGA tumors. Via the “mutations” module, the *SPON2* altered site data may be shown in protein structure schematics or three-dimensional (3D) structures. The “Comparison” module was also used to gather information on the variations in overall, disease-free, progression-free, and disease-free survival in TCGA cancer patients with or without a genetic mutation in *SPON2*. Moreover, log-rank *P* values for Kaplan–Meier plots were constructed.

### 2.4. Immunohistochemistry (IHC) Staining

We downloaded IHC images of *SPON2* protein expression in normal tissues and 6 tumors tissues from the HPA (Human Protein Atlas) (http://www.proteinatlas.org/) and analyzed, in order to facilitate the assessment of differences in *SPON2* expression at the protein level. These include LUAD (Lung Adenocarcinoma), BRCA (Breast invasive carcinoma), OV (Ovarian Serous Cystadenocarcinoma), LIHC (Liver hepatocellular carcinoma), TGCT (Testicular germ cell tumors), and THCA (Thyroid carcinoma).

### 2.5. Immune infiltration analysis

We investigated the association between the expression of *SPON2* and immune infiltration in all TCGA cancers using the “immune gene” module of the TIMER2 web server. We chose tumor-associated fibroblasts and T cells for additional investigation. To estimate immune infiltration, we used the TIMER, CIBERSORT, CIBERSORT-ABS, QUANTISEQ, XCELL, MCPCOUNTER, and EPIC algorithms. The purity-adjusted Spearman grade correlation test was used to determine *P* values and partial correlation (cor) values.^[26]^ The data were visualized as a heatmap and a scatter plot.

### 2.6. *SPON2*-related gene enrichment analysis

Then, we searched the STRING website (https://string-db.org) using the terms “Human Sapiens” and “*SPON2*,” a single protein. Subsequently, we set the following main parameters: minimum required interaction score [“Low confidence (0.150)”], meaning of network edges “evidence,” max number of interactors to show (“no more than 50 interactors” in 1st shell and “no more than 50 interactors” in 2nd shell) and active interaction sources “experiments.” Ultimately, the *SPON2*-binding proteins that have been experimentally determined were retrieved.

Based on datasets of all TCGA cancers and normal tissues, we used the “Similar Gene Detection” module of GEPIA2 to retrieve the top 100 *SPON2*-correlated targeting genes. We also carried out a pairwise gene Pearson correlation study of *SPON2* and certain genes using the “correlation analysis” module of GEPIA2. For the dot plot, the log2 TPM was used. There were indications of the *P* value and the correlation coefficient (R). Also, we utilized TIMER2’s “Gene Corr” module to provide the heatmap data of the chosen genes, which includes the partial correlation (cor) and *P* value in the purity-adjusted Spearman’s rank correlation test.^[27]^

In addition, we merged the 2 types of data to run the KEGG pathway analysis (Kyoto encyclopedia of genes and genomes). The “tidy” and “ggplot2” R packages were used to eventually illustrate the enriched paths. This study was conducted using the R language program [*R*-3.6.3, 64-bit] (https://www.r-project.org/).^[28]^ *P* < .05 with Two-tailed. was regarded as statistically significant.

## 3. Results

### 3.1. *SPON2* expression is upregulated in multiple tumors

**Figure 1.**
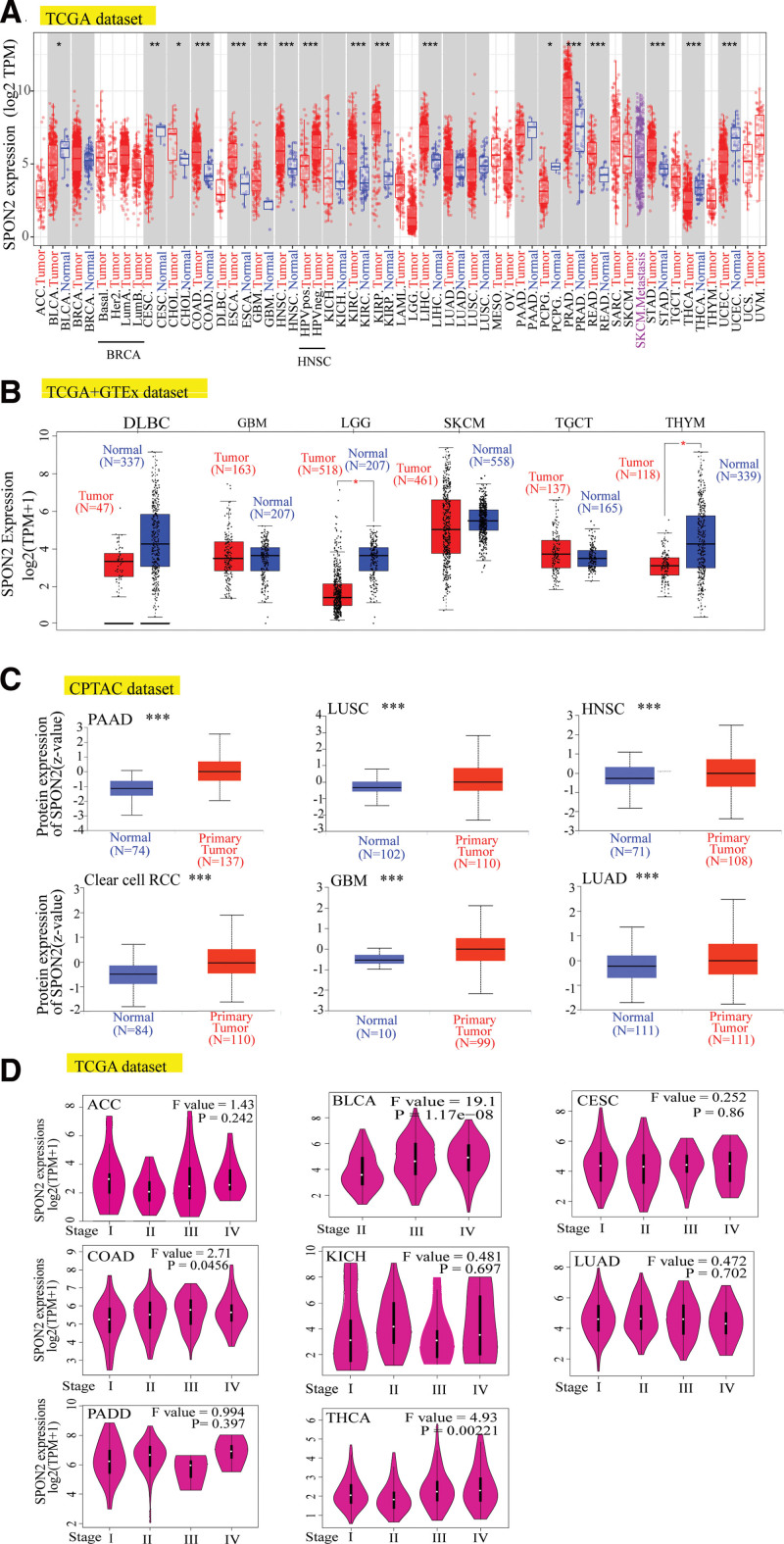
Expression level of *SPON2* gene in different tumors and pathological stages. (A) The expression status of the *SPON2* gene in different cancers or specific cancer subtypes was analyzed through TIMER2. *P < .05; **P < .01; ***P < .001. (B) For the type of DLBC (Tumor N = 47; Normal N = 337), GBM (Tumor N = 163; Normal N = 207), LGG (Tumor N = 518; Normal N = 207), SKCM (Tumor N = 461; Normal N = 558), TGCT (Tumor N = 137; Normal N = 165), and THYM (Tumor N = 118; Normal N = 339) in the TCGA project, the corresponding normal tissues of the GTEx database were included as controls. The box plot data were supplied. **P < .01. (C) Based on the CPTAC dataset, we also analyzed the expression level of *SPON2* total protein between normal tissue and primary tissue of PAAD (Tumor N = 74; Normal N = 137), LUSC (Tumor N = 102; Normal N = 110), HNSC (Tumor N = 71; Normal N = 108), ccRCC (Tumor N = 84; Normal N = 110), GBM (Tumor N = 10; Normal N = 99) and LUAD (Tumor N = 111; Normal N = 111). ***P < .001. (D) Based on the TCGA data, the expression levels of the *SPON2* gene were analyzed by the main pathological stages (stage I, stage II, stage III, and stage IV) of ACC, BLCA, CESC, COAD, KICH, LUAD, PAAD, and THCA. Log2 (TPM + 1) was applied for log-scale. ACC = adrenocortical carcinoma, BLCA = bladder urothelial carcinoma, ccRCC = clear cell renal cell carcinoma, COAD = colon adenocarcinoma, DLBC = Lymphoid neoplasm diffuse large B-cell lymphoma, GBM = glioblastoma multiforme, KICH = kidney chromophobe, LGG = brain lower grade glioma, LUAD = lung adenocarcinoma, LUSC = lung squamous cell carcinoma, PAAD = pancreatic adenocarcinoma, STAD = stomach adenocarcinoma, TCGA = The Cancer Genome Atlas, TGCT = testicular germ cell tumors, THCA = thyroid carcinoma, THYM = Thymoma.

To determine the features of *SPON2* mRNA expression, we combined tumor and normal samples from TCGA datasets. Figure 1A illustrates the expression of *SPON2* in tumor tissues from various cancers, including colon adenocarcinoma (COAD), esophageal carcinoma, head and neck squamous cell carcinoma (HNSC), kidney renal clear cell carcinoma (KIRC), LIHC, prostate adenocarcinoma (PRAD), rectum adenocarcinoma, stomach adenocarcinoma (STAD), kidney renal papillary cell carcinoma (KIRP), thyroid carcinoma (THCA) (*P* < .001), cervical squamous cell carcinoma and endocervical adenocarcinoma (CESC), GBM (*P* < .01), Pheochromocytoma and Paraganglioma, bladder urothelial carcinoma(BLCA), and cholangitis carcinoma (*P* < .05) was higher than that of normal tissues, while the expression level of *SPON2* in the tumor tissues of Uterine Corpus Endometrial Carcinoma was lower than that in normal tissues.

With the inclusion of the GTEx dataset’s normal tissues as controls, we further assessed how the expression of *SPON2* varied across both benign and cancerous tissues. While *SPON2* was expressed at lower levels in the tumor tissues of Lymphoid neoplasm diffuse large B-cell lymphoma and THYM than in normal tissues, we discovered that GBM, skin cutaneous melanoma, brain lower grade glioma (LGG), and TGCT showed greater expression in tumor tissues (Fig. 1B, *P* < .01). The *SPON2* total protein was more highly expressed in PAAD, LUSC, HNSC, ccRCC, GBM, and LUAD tissues than in normal tissues, as demonstrated by the CPTAC dataset results (Fig. 1C, *P* < .001). Meanwhile, ACC, BLCA, CESC, COAD, KICH, LUAD, PAAD, and THCA were among the cancers whose pathological stages were correlated with *SPON2* expression using the “Pathological Stage Plot” module of HEPIA2 (Fig. 1D, all *P* < .05).

### 3.2. *SPON2* expression is related to the prognosis of various tumors

**Figure 2.**
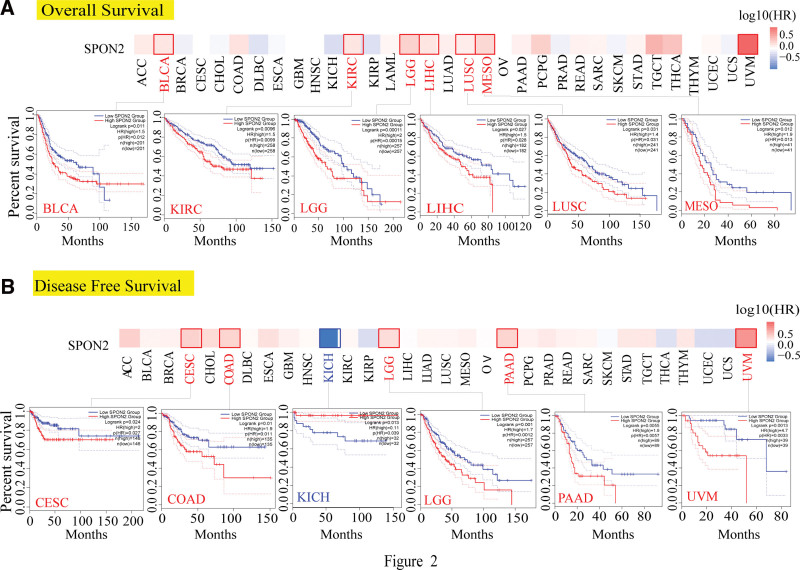
Correlation between *SPON2* gene expression and survival prognosis of cancers in TCGA. We used the GEPIA2 tool to perform overall survival (A) and disease-free survival (B) analyses of different tumors in TCGA by *SPON2* gene expression. The survival map and Kaplan–Meier curves with positive results are given. Red label indicated positively correlated, blue label indicated negatively correlated. ACC = adrenocortical carcinoma, BLCA = bladder urothelial carcinoma, BRCA = breast invasive carcinoma, CESC = cervical squamous cell carcinoma and endocervical adenocarcinoma, CHOL = cholangio carcinoma, COAD = colon adenocarcinoma, DLBC = Lymphoid neoplasm diffuse large B-cell lymphoma, ESCA = esophageal carcinoma, GBM = glioblastoma multiforme, HNSC = head and neck squamous cell carcinoma, KICH = kidney chromophobe, KIRC = kidney renal clear cell carcinoma, KIRP = kidney renal papillary cell carcinoma, LGG = brain lower grade glioma, LIHC = liver hepatocellular carcinoma, LUAD = lung adenocarcinoma, LUSC = lung squamous cell carcinoma, MESO = mesothelioma, OV = ovarian serous cystadenocarcinoma, PAAD = pancreatic adenocarcinoma, PCPG = pheochromocytoma and Paraganglioma, PRAD = prostate adenocarcinoma, READ = rectum adenocarcinoma, SKCM = skin cutaneous melanoma, STAD = stomach adenocarcinoma, TCGA = The Cancer Genome Atlas, TGCT = testicular germ cell tumors, THCA = thyroid carcinoma, THYM = Thymoma, UCEC = Uterine Corpus Endometrial Carcinoma, UVM = Uveal Melanoma.

To investigate the relationship between *SPON2* expression and the prognosis of patients with various cancers, we classified tumor cases into 2 groups based on the degree of *SPON2* expression: a high-expression group and a low-expression group. As shown by Figure 2A, the TCGA project found a correlation between highly expressed *SPON2* and a poor OS prognosis for the cancer types BLCA (*P* = .011), KIRC (*P* = .0096), LGG (*P* = .00011), LIHC (*P* = .027), LUSC (*P* = .031), and mesothelioma (*P* = .012). Depending on the DFS analysis results (Fig. 2B), the TCGA cases of CESC (*P* = .024), COAD (*P* = .01), LGG (*P* = .001), PAAD (*P* = .0055), and Uveal Melanoma (*P* = .0013) all had poor prognoses. Likewise, a poor DFS prognosis for KICH was associated with decreased *SPON2* gene expression (Fig. 2B, *P* = .013).

### 3.3. The characteristics of *SPON2* mutations in the TCGA pan-cancer cohort

**Figure 3.**
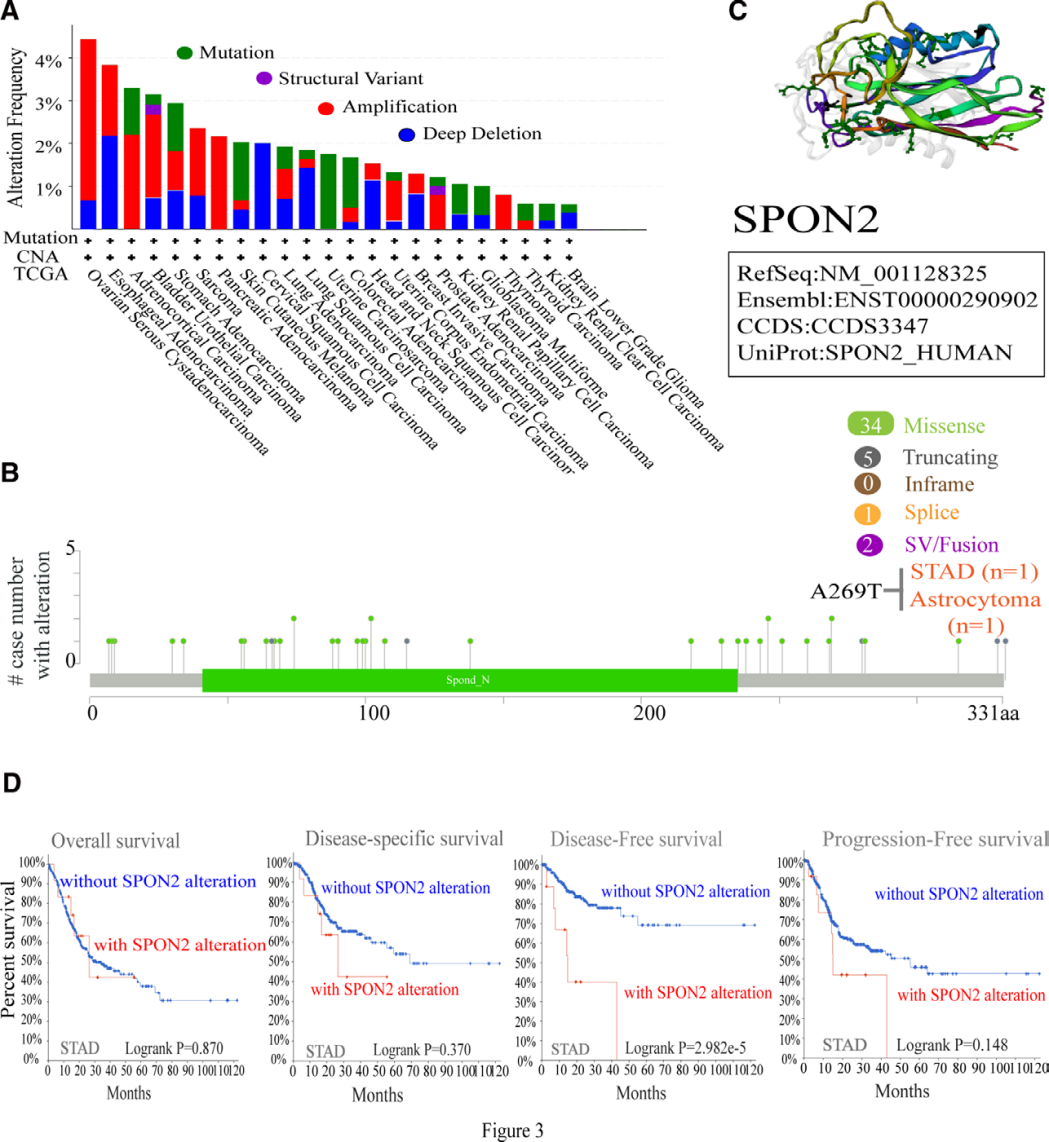
Mutation feature of *SPON2* in different tumors of TCGA. We analyzed the mutation features of *SPON2* for the TCGA tumors using the cBioPortal tool. The alteration frequency with mutation type (A) and mutation site (B) are displayed. We display the mutation site with the highest alteration frequency (A269T) in the 3D structure of *SPON2* (C). We also analyzed the potential correlation between mutation status and overall, disease-specific, disease-free and progression-free survival of STAD (D) using the cBioPortal tool. STAD = stomach adenocarcinoma, TCGA = The Cancer Genome Atlas.

We looked examined *SPON2’s* genetic status in several tumor samples from the TCGA cohorts. As shown by Figure 3A, the amplification is seen in patients with OV (Ovarian Cancer) tumors and has the greatest modification frequency of *SPON2* (−4%). Moreover, the Esophageal Adenocarcinoma revealed a copy number alteration frequency of > 3% and was primarily composed of the “Deep Deletion” alteration type (Fig. 3A). Additionally, the PAAD and THYM cases with genetic alterations showed amplification of *SPON2*, whereas all UCS cases with genetic alterations showed mutations in *SPON2* (Fig. 3A). The types, sites, and case numbers of the *SPON2* genetic mutation are also shown in Figure 3B. Missense mutations were more common in *SPON2* than other types of genetic alterations. Besides this, *SPON2* gene missense mutations caused by A269T changes were found in 1 instance of Astrocytoma and 1 case of STAD (Fig. 3b). The *SPON2* 3D structure is displayed in Figure 3C. The clinical survival prognosis of patients with all forms of cancer was not shown to be correlated with genetic *SPON2* changes. Instances of the STAD findings are displayed in Figure 3D.

### 3.4. Genetic immunohistochemical analysis data visually showed the expression of *SPON2* at the protein level.

**Figure 4.**
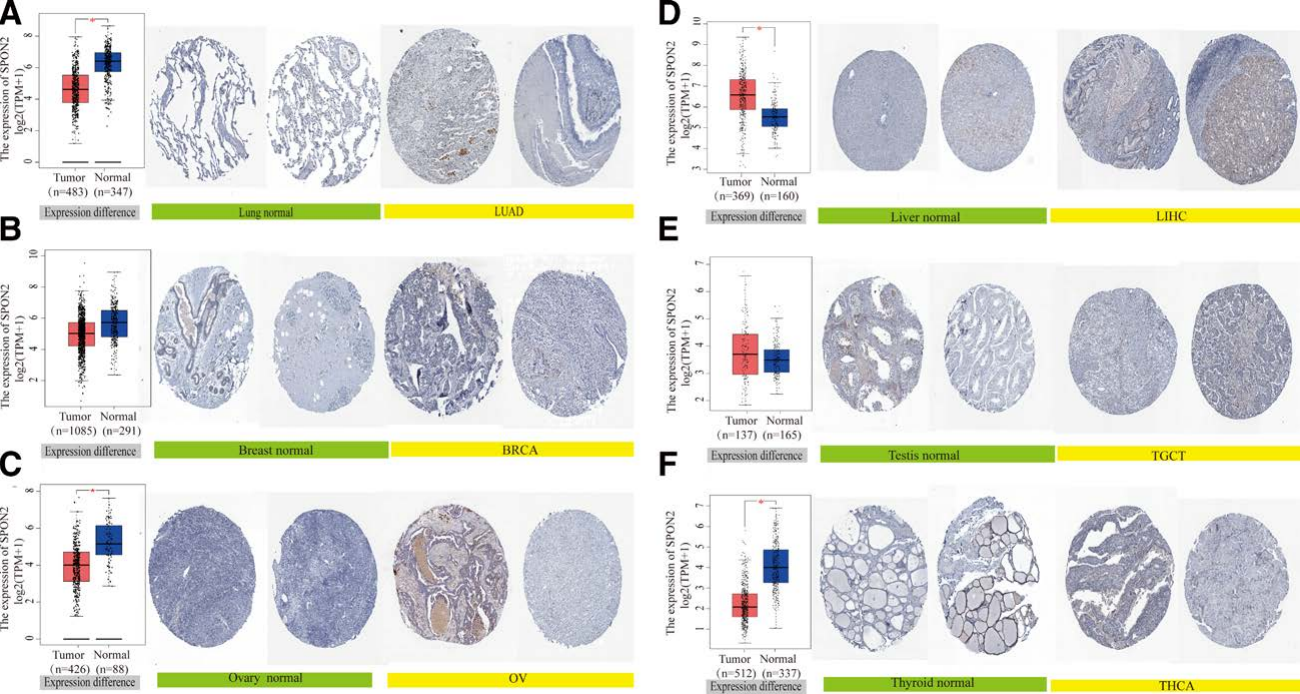
Gene expression and immunohistochemistry of *SPON2* in tumors and normal tissues. For the type of LUAD, BRCA, OV, LIHC, TGCT, and THCA in the TCGA project, the corresponding normal tissues of the GTEx database were included as controls. The box plot data were supplied. * *P < *.05. We also used the HPA database to obtain immunohistochemical results of *SPON2* in tumors and normal tissues. The protein expression of GPC2 in immunohistochemical images of normal (left) and tumor (right) groups. (A) Lung. (B) Breast. (C) Ovary. (D) Liver. (E) Testis. (F) Thyroid. BRCA = breast invasive carcinoma, LIHC = liver hepatocellular carcinoma, LUAD = lung adenocarcinoma, OV = ovarian serous cystadenocarcinoma, TCGA = The Cancer Genome Atlas, TGCT = testicular germ cell tumors, THCA = thyroid carcinoma.

Figure 4 depicts our first assessment of the variation in *SPON2* expression between healthy and tumor tissues. We found that LIHC and TGCT had increased expression in tumor tissues, whereas *SPON2* had decreased expression in the LUAD, BRCA, OV, and THCA tumor tissues compared to normal tissues. Even, the results of the immunohistochemistry of the OV, LUAD, BRCA, LIHC, TGCT, and THCA tumor and normal tissues are better proof of this. Normal lung, breast, ovary, liver, testis, and thyroid tissues showed negative or moderate staining for TWF1 IHC, whereas tumor tissues showed moderate or strong staining. In normal lung tissue, there are a lot of red blood cells and alveolar capillaries. LUAD cells are visible with their loose chromatin and vesicular nuclei. A normal breast cell has a consistent size and shape, a reasonable amount of cytoplasm, and visible vacuoles. In rare instances, tumor cells significantly grow to form solid nests or nodules, and BRCA cells form tubular, fibroadenoma, or acinar-like forms. Normal ovarian cells have a single, nest-like, lamellar, papillary layout, whereas OV cells have a sieve-like, microencapsulated structure. Euchromatin was abundant in normal hepatocytes, and some of them possessed nuclei that were binucleate or polyploid. Some LIHC cells include lipid vacuoles, and malignancy typically has modest cell changes. Most testicular cells had cuboidal epithelium and slit-like spaces. In TGCT cells, fibrovascular septas were seen separating the cells into compact clusters and nests. A collection of lymphocytes was observed accumulating along the septum. A few follicular cells were large and polygonal, with distinct borders and a large nucleus, in normal thyroid cells. Follicles and papillae that are closely clustered together are a THCA symptom, and some tumors are invasive.

### 3.5. Immunoinfiltration analysis of *SPON2* might explain its influence on the prognosis and survival of cancer patients

**Figure 5.**
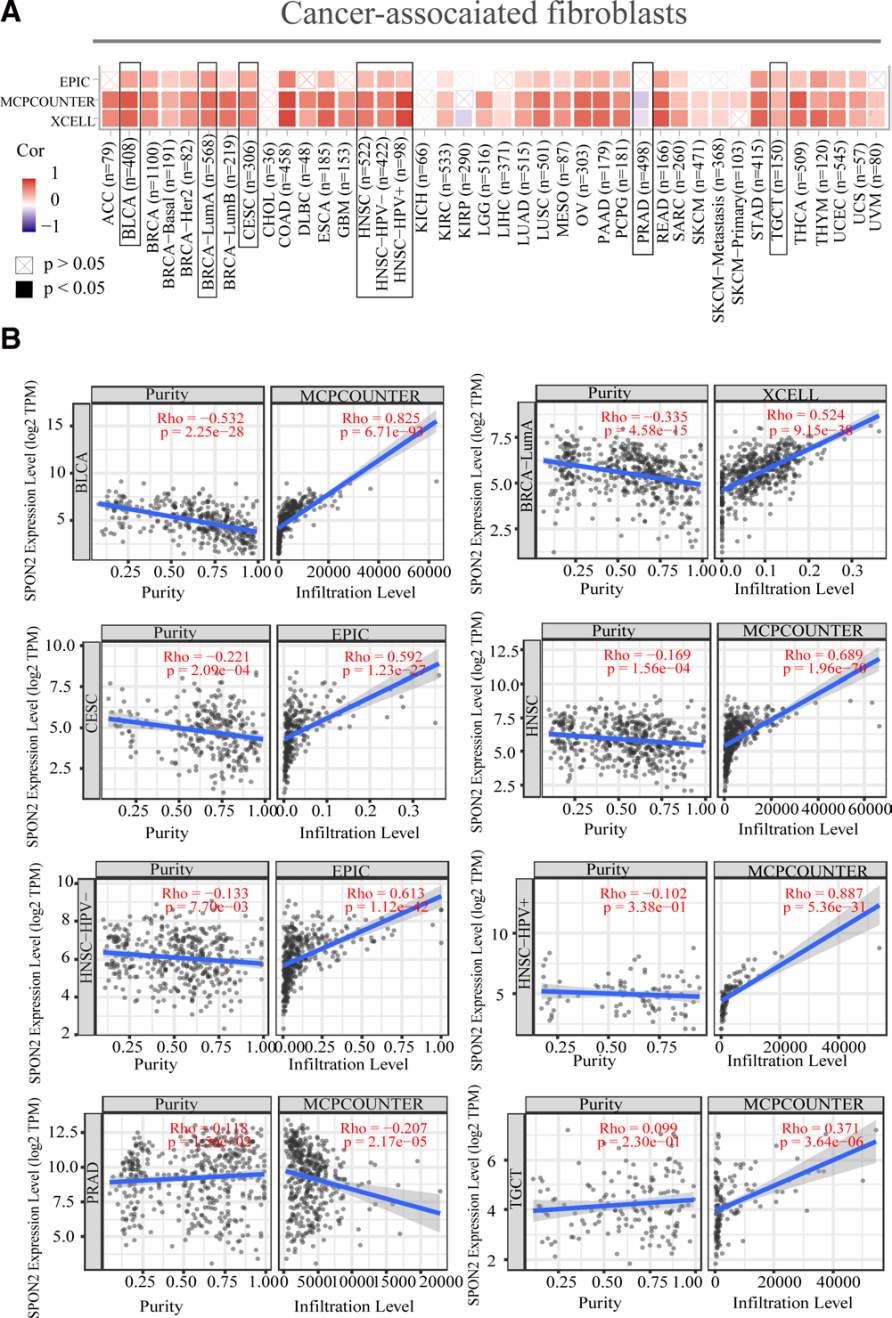
Correlation analysis between *SPON2* expression and immune infiltration of cancer-associated fibroblasts. Different algorithms (EPIC, MCPCOUNTER and XCELL) were used to explore the potential correlation between the expression level of the *SPON2* gene and the infiltration level of cancer-associated fibroblasts across all types of cancer in TCGA. (A) Represents the heat map of the correlation between *SPON2* and tumor-associated fibroblast infiltration. (B) This figure is a scatter plot representing the correlation between *SPON2* and tumor-associated fibroblast infiltration. ACC = adrenocortical carcinoma, BLCA = bladder urothelial carcinoma, BRCA = breast invasive carcinoma, CESC = cervical squamous cell carcinoma and endocervical adenocarcinoma, CHOL = cholangio carcinoma, COAD = colon adenocarcinoma, DLBC = Lymphoid neoplasm diffuse large B-cell lymphoma, ESCA = esophageal carcinoma, GBM = glioblastoma multiforme, HNSC = head and neck squamous cell carcinoma, KICH = kidney chromophobe, KIRC = kidney renal clear cell carcinoma, KIRP = kidney renal papillary cell carcinoma, LGG = brain lower grade glioma, LIHC = liver hepatocellular carcinoma, LUAD = lung adenocarcinoma, LUSC = lung squamous cell carcinoma, MESO = mesothelioma, OV = ovarian serous cystadenocarcinoma, PAAD = pancreatic adenocarcinoma, PCPG = pheochromocytoma and Paraganglioma, PRAD = prostate adenocarcinoma, READ = rectum adenocarcinoma, SKCM = skin cutaneous melanoma, STAD = stomach adenocarcinoma, TCGA = The Cancer Genome Atlas, TGCT = testicular germ cell tumors, THCA = thyroid carcinoma, THYM = Thymoma, UCEC = Uterine Corpus Endometrial Carcinoma, UVM = Uveal Melanoma.

The estimated filtration values of cancer-associated fibroblasts were found to be statistically positively correlated with *SPON2* expression in TCGA tumors of BLCA, CESC, BRCA-LumA, TGCT, HNSC, and HNSC [Human papillomavirus (Human papillomavirus) +/−], as shown in Figure 5. However, we observed an inverse correlation between PRAD and TGCT (Fig. 5). Figure 5 displays the scatterplot data for the tumors mentioned above that were produced by an algorithm. For instance, the MCPCOUNTER algorithm-based *SPON2* expression level in PRAD was inversely connected with the quantity of cancer-associated fibroblast infiltration (Rho = −0.207, *P* = 2.17e − 05). *SPON2* expression level based on MCPCOUNTER algorithm in BLCA (Rho = −0.825, *P* = 6.71e − 93), HNSC (Rho = 0.689, *P* = 1.96e − 70), HNSC-HPV + (Rho = 0.887, *P* = 5.36e − 31) and TGCT (Rho = 0.371, *P* = 3.64e − 06), XCELL algorithm in BRCA-LumA (Rho = 0.524, *P* = 9.15e − 38) and EPIC algorithm in CESC (Rho = 0.592, *P* = 1.23e − 27) and HNSC-HPV (Rho = 0.613, *P* = 1.12e − 42) were positively correlated with the number of cancer-associated fibroblast infiltration.

### 3.6. Enrichment analysis suggests that the “alcohol metabolic process” might be involved in the effect of *SPON2* on tumor pathogenesis

**Figure 6.**
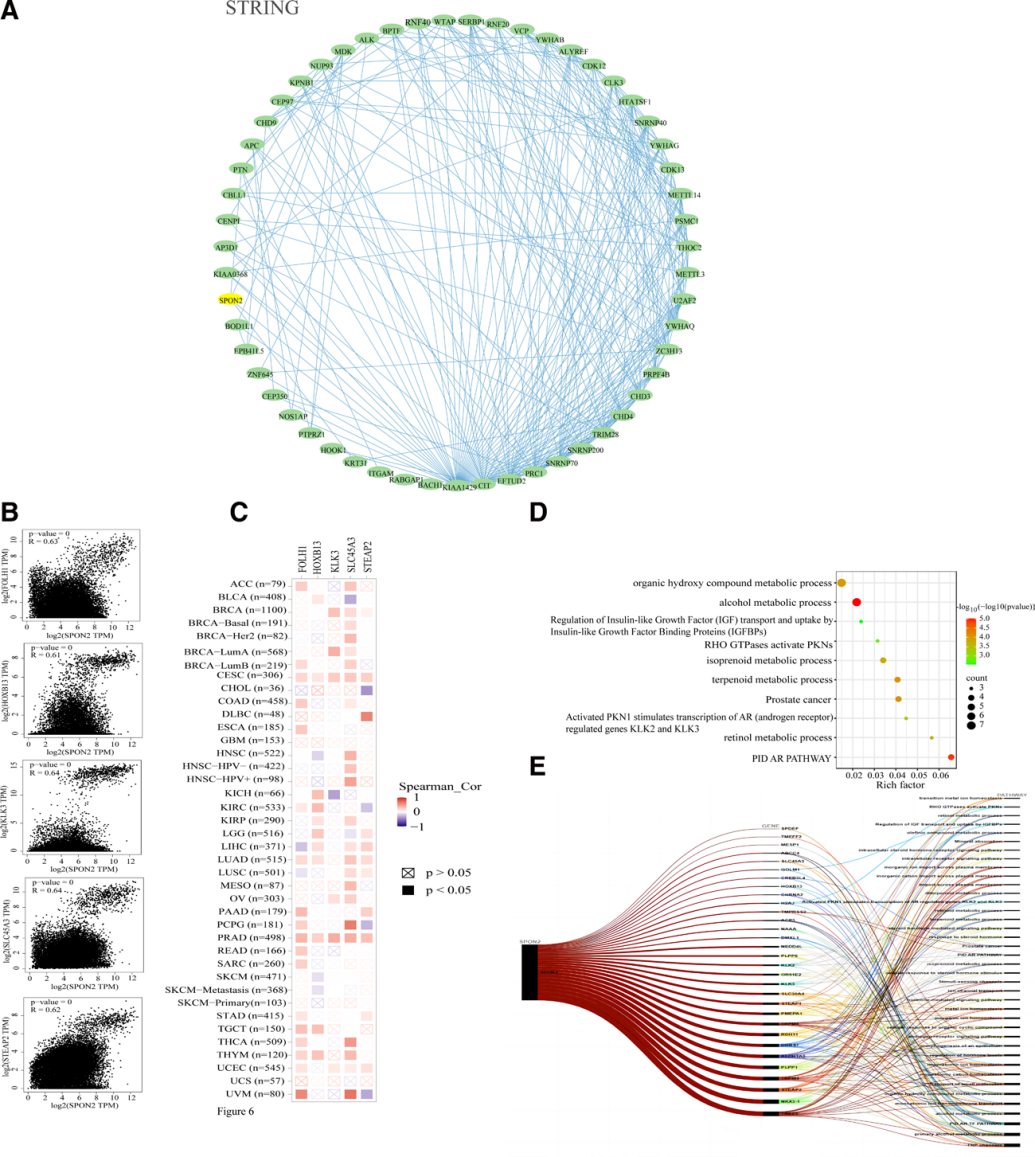
*SPON2*-related gene enrichment analysis. (A) We first obtained the available experimentally determined *SPON2*-binding proteins using the STRING tool. (B) Using the GEPIA2 approach, we also obtained the top 100 *SPON2*-correlated genes in TCGA projects and analyzed the expression correlation between *SPON2* and selected targeting genes, including SLC45A3, KLK3, FOLH1 and STEAP2. (C) The corresponding heatmap data in the detailed cancer types are displayed. (D) Based on the *SPON2*-binding and interacted genes, KEGG pathway analysis was performed. (E) Sankey diagram of *SPON2*: The picture demonstrates the pan-oncogene and pathway associated with *SPON2* gene. ACC = adrenocortical carcinoma, AR = androgen receptor, BLCA = bladder urothelial carcinoma, BRCA = breast invasive carcinoma, CESC = cervical squamous cell carcinoma and endocervical adenocarcinoma, CHOL = cholangio carcinoma, COAD = colon adenocarcinoma, DLBC = Lymphoid neoplasm diffuse large B-cell lymphoma, ESCA = esophageal carcinoma, GBM = glioblastoma multiforme, HNSC = head and neck squamous cell carcinoma, IGF = insulin-like growth factor, IGFBPs = insulin-like growth factor binding proteins, KICH = kidney chromophobe, KIRC = kidney renal clear cell carcinoma, KIRP = kidney renal papillary cell carcinoma, LGG = brain lower grade glioma, LIHC = liver hepatocellular carcinoma, LUAD = lung adenocarcinoma, LUSC = lung squamous cell carcinoma, MESO = mesothelioma, OV = ovarian serous cystadenocarcinoma, PAAD = pancreatic adenocarcinoma, PCPG = pheochromocytoma and Paraganglioma, PRAD = prostate adenocarcinoma, READ = rectum adenocarcinoma, SKCM = skin cutaneous melanoma, STAD = stomach adenocarcinoma, TCGA = The Cancer Genome Atlas, TGCT = testicular germ cell tumors, THCA = thyroid carcinoma, THYM = Thymoma, UCEC = Uterine Corpus Endometrial Carcinoma, UVM = Uveal Melanoma.

We sought to screen specific *SPON2*-binding proteins and genes whose expression is connected with *SPON2* for a series of pathway enrichment studies to better study the molecular mechanism of the *SPON2* gene in carcinogenesis. We identified a total of 54 *SPON2*-binding proteins using the STRING program, all of which have experimental support. The interaction network of these proteins is shown in Figure 6A. The top 100 genes that linked with *SPON2* expression were generated by combining all of the TCGA tumor expression data using the GEPIA2 program. As shown in Figure 6B, there was a significant correlation between the expression levels of *SPON2* and *FOLH1* (folate hydrolase 1), *HOXB13* (homeobox B13), *KLK3* (kallikrein-3), *SLC45A3* (Solute Carrier Family 45 Member 3), and *STEAP2* (The 6-transmembrane epithelial antigen of prostate 2) (*R* = 0.63, *R* = 0.61, *R* = 0.64, *R* = 0.64, and *R* = 0.62, all genes *P* < .001). In the majority of specific cancer types, the related heat map data also demonstrate a favorable association between *SPON2* and the aforementioned 5 genes (Fig. 6C). Furthermore, The 2 datasets were also integrated for KEGG enrichment analysis. The KEGG data in Figure 6D suggest that the “alcohol metabolic process” might be involved in the effect of *SPON2* on tumor pathogenesis.

Concurrently, The top 10 genes with high correlation between *SPON2* monogenes and other pan-oncogenes were TRPV6, NKX3-1, STEAP2, TRPM4, PLPP1, ALDH1A3, DHRS7, RDH11, TRPM8, and PMEPA1.TRP channels, Primary alcohol metabolic process, PID AR TF pathway, Alcohol metabolic process, Monoatomic ion transmembrane transport, Organic hydroxy compound metabolic process, Transport of small molecules, Monoatomic cation homeostasis, Monoatomic ion homeostasis, Regulation of hormone levels were the top ten pathways with high correlation between *SPON2* monogene and other pan-cancer pathways (Fig. 6E).

## 4. Discussion

*SPON2* is expressed in a variety of cell types and plays a role in cell adhesion, migration, and differentiation.^[3,21,29,30]^ It is a host innate immune regulator, which represents a unique pattern-recognition molecule in the ECM for microbial pathogens.^[31]^ Notably, *SPON2* also acts as an integrin-ligand for inflammatory cell recruitment and T cell activation.^[32,33]^ The binding of *SPON2* to bacteria promotes phagocytosis in bacteria and stimulates macrophages to produce pro-inflammatory cytokines.^[34]^ There were no pan-cancer studies of *SPON2* that we could locate in the literature. Consequently, using the TCGA, CPTAC, and GEO datasets, we found the *SPON2* gene in distinct cancers.

In our study, *SPON2* was overexpressed in the majority of tumor tissues as compared to normal tissues. Also, by examining the *SPON2* gene’s survival and prognosis, we have drawn various findings for various tumor types. The findings demonstrated a negative correlation between high *SPON2* expression in LGG patients and poor OS prognosis (*P* = .00015), poor DFS *P* = .0012). Unfortunately, there aren’t many reports of *SPON2*’s function in LGG tumors. These findings might lead to the development of a fresh clinical biomarker for the prognosis of LGG patients.

About stomach adenocarcinoma, we found a correlation between high expression of *SPON2* and poor OS prognosis (*P* = .23) and poor DFS (*P* = .076). Kuramitsu et al^[35]^ reported that cancer-associated fibroblast-derived *SPON2* might promote peritoneal dissemination (PD) in part by promoting the motility of GC cells and serving as a prognostic marker of PD in GC. The results of Lu et al^[36]^ showed that *SPON2* was highly expressed in gastric cancer tissues of patients with recurrence or metastasis. Down-regulation of *SPON2* inhibits the cell cycle of first gap/synthesis (G1/S), accelerates apoptosis through the mitochondrial pathway, and inhibits epithelial-mesenchymal conversion by blocking the activation of the extracellular regulated protein kinases1/2 (ERK1/2) pathway. Jin et al^[7]^ revealed the expression of spondin-2 was up-regulation in patients with depth of invasion (T3/T4) and lymph node metastasis (N1-3) indicating higher invasive and metastasizing activity in spondin-2 high-expression cancer cells. In addition, spondin-2 was positively correlated with MMP-9 protein expression in gastric cancer. Kang et al^[37]^ show that the expression and functioning of *SPON2* is regulated by Notch signaling pathway via RBP-Jk transcription factor. In summary, *SPON2* plays a carcinogenic role in the development of gastric cancer and may be used as a marker for gastric cancer diagnosis and a new therapeutic target for gastric cancer.

According to our analysis, individuals with LIHC who had high *SPON2* expression had poor OS. According to research by Feng et al,^[38]^ the increase of *SPON2* protein expression in hepatocellular carcinoma (HCC) tissues was strongly linked to a decline in OS and DFS following radical resection. As per Yan-Li Zhang et al (2018), *SPON2* has the dual role of enhancing the invasion of m1-like macrophages and preventing tumor metastasis in the tumor microenvironment of HCC. The *SPON2*-a4b1 integrin signaling pathway upregulates F-actin recombination, activates Rac1 and RhoA, and encourages the attraction of m1-like macrophages.^[39]^ Research has revealed that the thyroid hormone-regulated protein *SPON2* inhibits the migration and invasion of HCC cells.^[20]^ These findings demonstrate that *SPON2* is a key factor in mediating the immune response in HCC against tumor cell growth and migration.^[21]^

We discovered a connection between high *SPON*2 expression and a poor DFS prognosis for colon cancer (*P* = .011). According to Zhang et al,^[40]^ *SPON2* protein expression was substantially connected with age and the M phase, and its expression was significantly correlated with the CRC stage, T phase, M phase, and Dukes phase. According to Huang et al,^[41]^ *SPON2* stimulated the integrated 1/PYK2 axis, promoting cytoskeletal remodeling and transendothelial migration of monocytes. The development and spread of colorectal cancer tumors are significantly influenced by *SPON2*-driven M2-TAM expansion. There are findings show that CF-10 may be a successful candidate for treating recalcitrant CRCs and potential to overcome 5-FU resistance.^[42]^ The possible effect of *SPON2* on the prognosis of colorectal adenocarcinoma needs further study.

In addition, Hu et al^[43]^ shows that upregulation of *SPON2* in TNBC is associated with poorer patient outcomes. Knockdown of *SPON2* up-regulated TNBC cell adhesion and down-regulated PI3K-ATK pathway, and PPI results showed that CCL2 was the key protein. There were results suggested that a novel combination of miR-214-5P and olaparib could be an effective therapy for BRCA-wild-type TNBCs and reduce racial disparities in TNBC outcomes.^[44]^ *SPON2* intervention can be further studied. Ming Wu et al(2023) found that *SPON2* promotes the bone metastasis of lung adenocarcinoma via activation of the NF-κB signaling pathway.^[45]^ According to Chen et al,^[46]^ Tumor-derived exosomes HOTAIRM1 can transfer to CAFs, regulate the expression of *SPON2* in CAFs cells, and ultimately promote the progression of NSCLC. According to Ni et al,^[47]^ *SPON2* silencing inhibited the proliferation of LSCC cells through inhibiting the activation of PI3K/AKT signaling. Taken together, Many studies have shown that *SPON*2 may be a latent prognostic biomarker for clinical diagnosis and assessment of cancers.

These findings have important clinical implications that may affect patient care. *SPON2* can be used as a prognostic marker, therapeutic target or therapeutic response indicator, but further studies such as in vitro or in vivo experiments are needed to further clarify the biological significance of *SPON2* and verify its potential application in the clinical setting. These findings may help elucidate the role of *SPON2* in tumorigenesis and progression and provide a reference for achieving more precise and personalized immunotherapy in the future.

## 5. Conclusions

To sum up, our results indicate that *SPON2* is aberrantly expressed in various cancers and is significantly associated with the prognosis of cancer patients. *SPON2* alterations, including mutations, duplications and amplifications, were identified in multiple cancer types. The expression of *SPON2* is significantly correlated with the infiltration of immune cells into the tumor microenvironment and the immunotherapy response of various cancers. The comprehensive pan-cancer analysis we performed helps to elucidate the involvement of *SPON2* in tumorigenesis from multiple perspectives, suggesting that *SPON2* may be a potential prognostic biomarker for clinical diagnosis and assessment of cancer

## Author contributions

**Conceptualization:** Jiali Tang, Shinong Gu.

**Data curation:** Shinong Gu, Qing Huang.

**Formal analysis:** Shinong Gu, Qing Huang.

**Funding acquisition:** Shinong Gu.

**Investigation:** Shinong Gu, Qing Huang.

**Methodology:** Jiali Tang, Shinong Gu, Qing Huang.

**Project administration:** Qing Huang.

**Resources:** Shinong Gu, Qing Huang.

**Software:** Shinong Gu, Xuanwen Li.

**Supervision:** Jiali Tang.

**Validation:** Shinong Gu, Qing Huang.

**Visualization:** Jiali Tang.

**Writing – original draft:** Jiali Tang, Xuanwen Li.

**Writing – review & editing:** Xuanwen Li.
